# Novel Molecule Nell-1 Promotes the Angiogenic Differentiation of Dental Pulp Stem Cells

**DOI:** 10.3389/fphys.2021.703593

**Published:** 2021-08-26

**Authors:** Mengyue Li, Qiang Wang, Qi Han, Jiameng Wu, Hongfan Zhu, Yixuan Fang, Xiuting Bi, Yue Chen, Chao Yao, Xiaoying Wang

**Affiliations:** ^1^Shandong Key Laboratory of Oral Tissue Regeneration, Shandong Engineering Laboratory for Dental Materials and Oral Tissue Regeneration, School and Hospital of Stomatology, Cheeloo College of Medicine, Shandong University, Jinan, China; ^2^Jinan Stomatological Hospital, Jinan, China

**Keywords:** human dental pulp cells, nel-like molecule-1, human umbilical vein endothelial cells, angiogenesis, pulp regeneration

## Abstract

**Introduction:**

This work aimed to reveal the crucial role of Nell-1 in the angiogenic differentiation of human dental pulp stem cells (DPSCs) alone or co-cultured with human umbilical vein endothelial cell (HUVECs) *in vitro* and whether this molecule is involved in the pulp exposure model *in vivo*.

**Methods:**

Immunofluorescence was conducted to ascertain the location of Nell-1 on DPSCs, HUVECs, and normal rat dental tissues. RT-PCR, Western blot, and ELISA were performed to observe the expression levels of angiogenic markers and determine the angiogenic differentiation of Nell-1 on DPSCs alone or co-cultured with HUVECs, as well as *in vitro* tube formation assay. Blood vessel number for all groups was observed and compared using immunohistochemistry by establishing a rat pulp exposure model.

**Results:**

Nell-1 is highly expressed in the nucleus of DPSCs and HUVECs and is co-expressed with angiogenic markers in normal rat pulp tissues. Hence, Nell-1 can promote the angiogenic marker expression in DPSCs alone and co-cultured with other cells and can enhance angiogenesis *in vitro* as well as in the pulp exposure model.

**Conclusion:**

Nell-1 may play a positive role in the angiogenic differentiation of DPSCs.

## Introduction

Nel-like molecule-1 (Nell-1) is a novel signaling molecule, with a crucial positive regulatory role in chondrogenesis and osteogenesis (Li C. et al., 2019); however, its involvement in pulp angiogenesis, which is an important part of pulp regeneration, remains poorly studied. Vasculogenesis is the differentiation of endothelial cells to form blood vessels during embryonic development, while angiogenesis means new vessels sprouting from pre-existing vasculature (Chung and Ferrara, 2011), both of them are mediated by angiogenic growth factors (Rombouts et al., 2017). Among which, vascular endothelial growth factor (VEGF) is a strong regulator of physiological and pathological angiogenesis during embryogenesis and pathological angiogenesis associated with tumors (Ferrara et al., 2003) and alveolar bone process morphogenesis (An et al., 2017). VEGF receptor (VEGFR) has a potential part in mediating the biological effects of VEGF, leading to homodimerization and autophosphorylation when VEGF dimers bind to VEGFR-1 and VEGFR-2 (Ferrara, 2002). Activating VEGFR-2 (Flk-1) could induce angiogenesis and increase vascular permeability, mitogenesis, and chemotaxis in endothelial cells.

Nell-1 encodes a secreted protein (Zhang et al., 2010) and was discovered by Ting et al. (1999), when they accidentally operated for the surgical correction of unilateral coronal synostosis. In our previous study, Nell-1 shows spatiotemporal expression patterns during murine tooth (Tang et al., 2013) and is mainly expressed in the odontoblasts, pulp fibroblasts, and endothelial cells of the blood vessels in human teeth (Tang et al., 2013; Liu et al., 2016). Fahmy-Garcia et al. (2018) confirmed that Nell-1 can enhance the migration of mesenchymal stem cells (MSCs) and the angiogenesis of HUVECs. DPSCs form a dentin/pulp-like complex (Gronthos et al., 2000) with neural-like cells (Stevens et al., 2008; Gonmanee et al., 2018; Li D. et al., 2019), endotheliocytes (d’Aquino et al., 2007), and vascular tissues (Karbanova et al., 2011) and have a predominant pro-angiogenic influence compared with dental follicle precursor cells (FSCs) (Hilkens et al., 2014). These data indicate that DPSCs are a promising population of stem cells that could achieve angiogenesis. The potential of Nell-1 to induce the angiogenetic differentiation of DPSCs is of great interest.

The survival rate of regenerating vascular dependent tissues could be increased when MSCs are co-transplanted with hematopoietic stem cells (Moioli et al., 2008). Endothelial cells (ECs) are a potential source of neovascularization during tissue regeneration (Moioli et al., 2008). Angiogenesis highly occurs between stem cells and endothelial cells through synergistic effect or direct cell contact (Aguirre et al., 2010; Allen et al., 2011; Rao et al., 2012; Kang et al., 2013). Several studies also confirmed that the co-culture of HUVECs with stem cells from the apical papilla (SCAPs) or DPSCs can enhance the angiogenic potential (Dissanayaka et al., 2012; Liu et al., 2018). Whether Nell-1 is directly involved in the angiogenetic differentiation of DPSCs co-cultured with HUVECs must be explored.

## Materials and Methods

### Isolation, Culture, and Identification of DPSCs and Co-culture of DPSCs With HUVECs

Third molars were acquired after obtaining informed consent from each patient (15–25 years of age, male and female) who underwent routine extraction with no caries or periodontal diseases. The extracted teeth were washed with PBS and cut with fissure in sterile conditions. The acquired pulp tissues were digested with 3% I-type collagenase (Solarbio, Beijing, China), and the dental tissues were transported in 25 cm^2^ cell culture flasks. HUVECs and its specific endothelial cell medium (ECM) were acquired from ScienCell company (San Diego, United States) (Leopold et al., 2019). Each cell type was used at passages 3–5 in all experiments.

Human DPSCs at passage 3 were collected. The cells were tested for MSC markers CD90, CD44 and CD105, and hematopoietic stem markers CD34 and CD45 by using flow cytometric (CytoFLEX, CA, United States) with PBS as the negative control. Osteogenic and adipogenic differentiation assays were performed on the DPSCs to detect their multi-lineage differentiation ability. The cells were stained and examined under an inverted microscope after 21 days. The experiments group was grown in osteogenic differentiation medium (α-MEM containing 10%FBS, 0.01 nmol/l dexamethasone, 10 mmol/l β-glycerophosphate, and 50 mg/mL ascorbic acid) (Sigma, St Louis, MO, United States) or adipogenic differentiation medium (Pythonbio, China), and the control group was treated similarly to the above cell culture.

DPSCs and HUVECs were mixed directly at a 1:1 number ratio with new mixed medium prepared by mixing the α-MEM (containing 10% FBS) with ECM at 1:1 ratio.

### RNA Extraction and Quantitative Real-Time Polymerase Chain Reaction (qRT-PCR)

DPSCs alone and co-culture groups were cultured in six-well plates in α-MEM (containing 10% FBS) or mixed medium with 0 and 50 ng/mL Nell-1 (CHO-derived human Nell-1 protein, R&D Systems, 5487-NL-050, Minneapolis, MN, United States) for 1, 2, 3, 7, and 14 days. Total RNA was isolated using Trizol (Takara, Tokyo, Japan). Isolated RNA sample of 1.0 μg weight was reverse-transcribed into cDNA with cDNA synthesis kit (Takara, Tokyo, Japan). qRT-PCR was performed with TB Green (Takara, Tokyo, Japan) and LightCycler 480 system. Primer sequences are shown in Table 1.

**TABLE 1:** Primer sequences used for real-time PCR.

**Gene**	**Forward sequences**	**Reverse sequences**
GAPDH	ATCACCATCTCCAGGAGCGA	CCTTCTCCATGGTGGTGAAGAC
VEGF	GAGCCTTGCCTTGCTGCTCTAC	CACCAGGGTCTCGATTGGATG
Flk-1	AGCCAGCTCTGGATTTGTGGA	CATGCCCTTAGCCACTTGGAA

### Western Blot

The cells were planked similarly to PCR, lysed with RIPA buffer containing 1% PMSF (Solarbio, Beijing, China) for 30 min. Cellular proteins were isolated and quantified by a BCA kit (Solarbio, Beijing, China), separated on 10% polyacrylamide gels, and transferred onto the polyvinylidene difluoride membranes (Millipore, Billerica, United States). Following washing with TBST, the membranes were incubated with primary antibodies including Flk-1 antibody (1:1,000 dilution; Novus, NB200-208, United States) and GAPDH antibody (1:10,000 dilution; Proteintech, China) overnight at 4°C. Then, the membranes were incubated with secondary antibodies for 2 h at RT and then washed again. The results were analyzed by Image J software.

### ELISA

Quantikine ELISA kit (Dakewe, Shenzhen, China) was used to measure VEGF expression, and the process was similar to that in RNA extraction. Cell supernatants were collected at 1, 2, 3, 7, and 14 days with 0 and 50 ng/mL Nell-1. ELISA was performed in accordance with manufacturer’s instructions.

### Cell Immunofluorescence for Nell-1

DPSCs and HUVECs were separately cultured in 24-well plates for 24 h. The cells were fixed with 4% paraformaldehyde and sealed with normal goat serum (Zhongshan, Beijing, China) at 37°C for 30 min, and then incubated with primary antibody for Nell-1 (1:100 dilution; Proteintech, China) at 4°C overnight. After being washed three times in PBS, the second antibodies (1:500 dilution; Zhongshan, Beijing, China) were incubated for 2 h. DAPI (Solarbio, Beijing, China) was used to stain the nucleus of pulp tissues. The cells were observed under a fluorescence microscope.

### *In vitro* Tube Formation Assay

To investigate which minimum concentration of Nell-1 could promote formation of endothelial tubules and a blood vessel network when DPSCs were co-cultured with HUVECs, Matrigel angiogenesis assay was carried out. Co-culture groups (HUVECs: DPSCs, 1:1) were cultured in 24-well plates in mixed medium (containing 0.5%FBS) with 0, 50, 100, and 200 ng/mL Nell-1 at 6 h. Images were observed under an inverted phase-contrast microscope.

### Tooth Sample Collection and Preparation

All animal experimental protocols were performed in accordance with the guidelines of the Institutional Review Board of School of Stomatology, Shandong University. Twelve Wistar rats were randomly categorized into three groups: (1) collagen group, pulp cavity was covered with collagen sponge (BIOT Biology, China) soaked with PBS; (2) Nell-1 group, pulp cavity was covered with collagen sponge soaked with 700 ng/mL Nell-1; and (3) normal teeth group, upper molars did not receive cavities or any other treatments. The occlusal surface of non-carious upper first molars was selected to establish the rat pulpitis model with a #35 K-file under anesthesia. Afterward, the cavities were sealed with glass ions (VOCO, Ionofil Molar, Germany) as the bottom material and with resin (3MESPE, Filtek Z350 XT, United States) for the collagen and Nell-1 groups. After 1 week, the rats were sacrificed. The experimental teeth were fixed in 4% paraformaldehyde for 24 h, demineralized with 10% ethylenediaminetetraacetic acid solution, and cut with a blade (5 um serial sections). The sections were processed for histological and immunohistochemical examinations.

### Histology, Immunohistochemistry, and Immunofluorescence

Hematoxylin and eosin (HE, Solarbio, Beijing, China) staining was used to observe the morphology and structure of pulp tissues.

Immunohistochemical staining and immunofluorescence were performed to observe the blood vessel count and distribution of Nell-1, VEGF, and Flk-1. Immunohistochemical analysis was performed by using the primary antibodies CD34 (absin, Shanghai, China) and CD31 (Servicebio, Wuhan, China), and the tissues were counterstained with hematoxylin. Double immunostaining of either VEGF (1:100 dilution; Novus, NB100-664SS, United States) or FLK-1 (1:100 dilution; Novus, NB200-208, United States) with Nell-1 (1:100 dilution; Proteintech, 21783-1-AP, China) and then immunostaining for second antibodies (1:500 dilution; absin, China) was performed to observe the relationship among Nell-1, VEGF, Flk-1. DAPI was used to stain the nucleus. Each slice was observed by a microscope (BX51, Olympus, Japan).

### Statistical Analyses

Student’s *t*-test or one-way ANOVA was used to compare statistical significance between various treatments and respective controls by using GraphPad Prism software. All experiments were repeated three times from independent samples from different donors. Data were expressed as mean ± standard deviation. *P* < 0.05 was considered statistically significant.

## Results

### Isolation and Characterization of DPSCs

**FIGURE 1:**
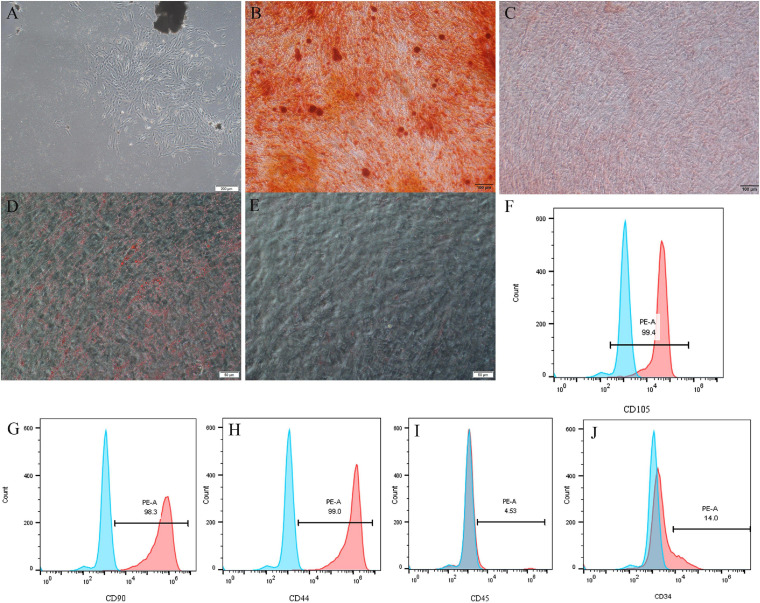
Isolation and characterization of DPSCs. **(A)** Representative image to display primary DPSCs. **(B–E)** DPSCs underwent osteogenic/odontogenic and adipogenic differentiation and were stained after 21 days. Alizarin red staining indicated mineralized nodules in the osteogenic/odontogenic group **(B)** but negative for the control group **(C)**. Oil red O images indicated oil droplets in the adipogenic group **(D)** but negative for the control group **(E)**. DPSCs were positive for mesenchymal stem cells markers CD105 **(F)**, CD90 **(G)**, and CD44 **(H)**, but negative for hematopoietic stem cells markers CD45 **(I)** and CD34 **(J)**.

The primary cells were successfully isolated from pulp tissues and displayed a long spindle shape with adherent growth (Figure 1A). Alizarin red and oil red O staining showed that DPSCs possess strong osteo/dentinogenic and adipogenic potential (Figures 1B–E). Flow cytometry results showed that DPSCs are positive for CD90, CD44, and CD105 but negative for CD34, CD45 (Figures 1F–J).

### Nell-1 Promoted the Angiogenesis-Related Gene and Protein Expression in DPSCs

**FIGURE 2:**
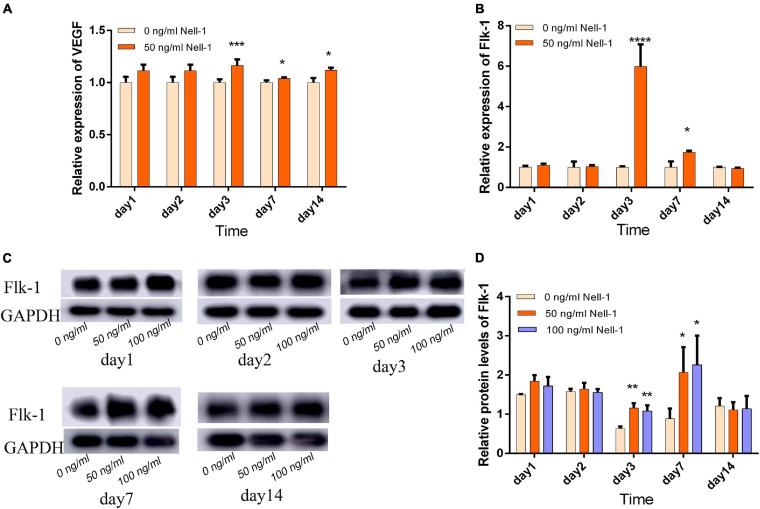
Angiogenesis-related gene and protein expression in DPSCs. **(A)** VEGF gene expression was increased by 50 ng/mL Nell-1 treatment in days 3, 7, and 14. **(B)** Flk-1 gene expression was increased by 50 ng/mL Nell-1 treatment in days 3 and 7. **(C,D)** Flk-1 protein expression was increased by 50 and 100 ng/mL Nell-1 treatment in days 3 and 7. Data are shown as the mean ± SD. (*p < 0.05, **p < 0.01, ***p < 0.001, ****p < 0.0001).

Angiogenic markers including VEGF and Flk-1 were detected by qRT-PCR. In DPSC group, 50 ng/mL Nell-1 remarkably promoted VEGF and Flk-1 expression compared with those in the control in days 3, 7, and 14 (apart from Flk-1 in day 14) (Figures 2A,B). The gene expression levels of VEGF in DPSC group increased in days 1 and 2 compared with those in the control group; however, no statistical significance was found.

Western blot analysis was performed to detect relative protein expression. In the experiment group, the angiogenesis-related protein expression was increased in days 3 and 7, but no significant difference was observed in days 1, 2, and 14 compared with those in the control group (Figures 2C,D).

### Nell-1 Promoted the Angiogenesis-Related Gene and Protein Expression and the Formation of Vessel-Like Structures in the Co-culture Group

**FIGURE 3:**
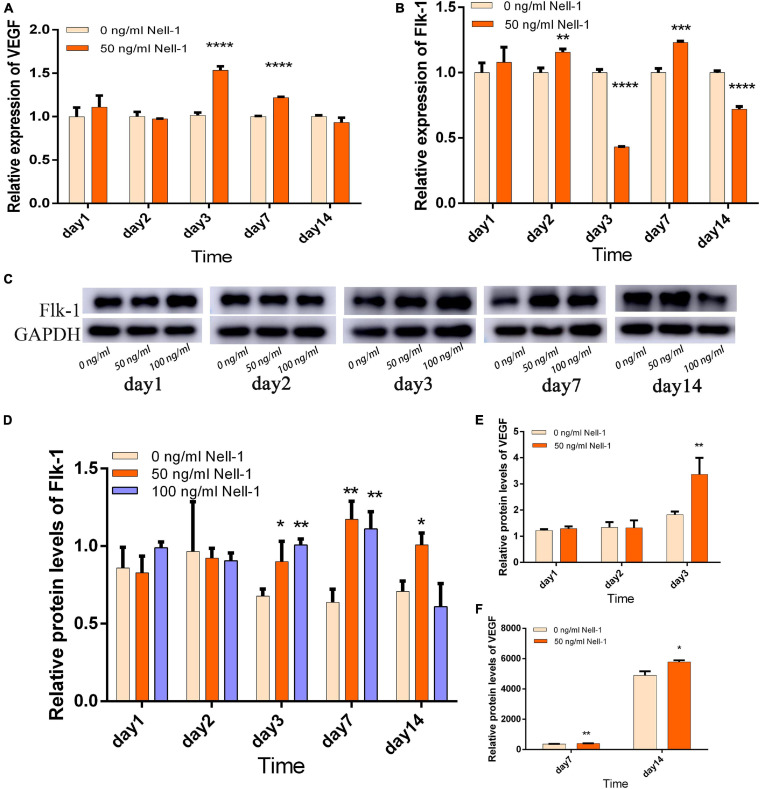
Angiogenesis-related gene and protein expression in the co-culture group. **(A)** VEGF gene expression was improved by 50 ng/mL Nell-1 treatment in days 3 and 7. **(B)** Flk-1 gene expression was improved by 50 ng/mL Nell-1 treatment in days 2 and 7, but was decreased in day 3 and 14. **(C,D)** Flk-1 protein expression was increased by 50 and 100 ng/mL Nell-1 treatment in days 3, 7, and 14. **(E,F)** VEGF protein expression was increased by 50 ng/mL Nell-1 treatment in days 3, 7, and 14. Data are shown as the mean ± SD. (*p < 0.05, **p < 0.01, ***p < 0.001, ****p < 0.0001).

On the basis of the VEGF expression levels, 50 ng/mL Nell-1 significantly promoted the angiogenetic differentiation in the experiment group compared with that in the control in days 3 and 7, but no statistical significance was observed in days 1, 2, and 14 (Figure 3A). Flk-1 expression level was increased by 50 ng/mL Nell-1 treatment compared with that in the control in days 2 and 7 (Figure 3B). However, these levels decreased significantly in days 3 and 14.

Western blot analysis and ELISA were performed to investigate Flk-1 and VEGF protein expression, respectively. In the experiment group, the angiogenesis-related protein Flk-1 was increased in days 3, 7, and 14, but no significant difference was found in days 1 and 2 compared with that in the control group (Figures 3C,D). The concentration of VEGF protein in supernatants secreted by the co-culture group was increased by 50 ng/mL Nell-1 compared with that in the control group in days 3, 7, and 14 (Figures 3E,F).

**FIGURE 4:**
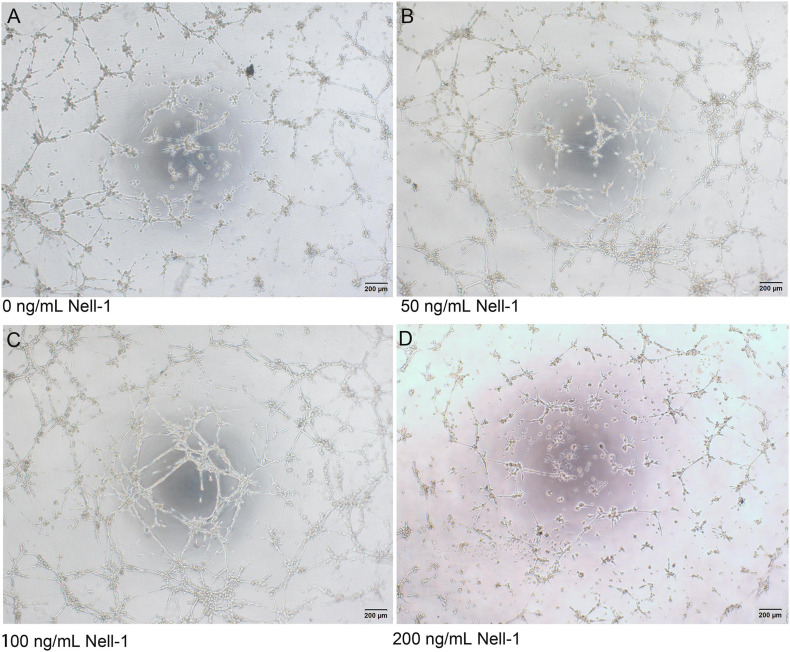
*In vitro* tube formation assay. **(A–D)** Phase-contrast images (× 40) of mixed cells with 0, 50, 100, and 200 ng/mL Nell-1 at 6h after seeding on Matrigel. Compared with control group **(A)**, 50 and 100 ng/mL Nell-1 group **(B,C)** showed more number of branching points and tubular length while 200 ng/mL Nell-1 group **(D)** had no significant changes.

Compared with control group, co-culture group seeded on Matrigel with 50 and 100 ng/mL Nell-1 formed more vessel-like structures, whereas no significant difference was observed in 200 ng/mL Nell-1 (Figure 4).

### Nell-1 Distribution in DPSCs, HUVECs, and Normal Pulp Tissues

**FIGURE 5:**
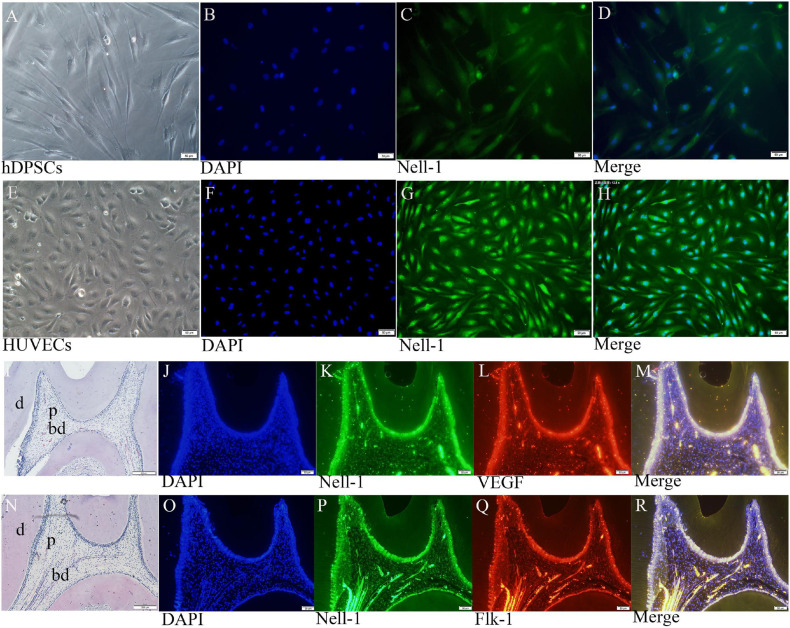
Nell-1 distribution in DPSCs **(A–D)**, HUVECs **(E–H)**, and normal pulp tissues **(I–R)**. **(A,E)** Normal cell feature of DPSCs and HUVECs. **(B,F)** DAPI: nucleus stain. **(C,G)** green fluorescence can be observed in the nucleus of DPSCs and HUVECs. **(D,H)** Merged images. **(I,N)** HE staining displayed the morphology and structure of normal rat pulp tissues. **(K,L,P,Q)** Nell-1, VEGF and Flk-1 can be found in odontoblasts, pulp fibroblasts, and endothelial cells of the blood vessels. **(J,O)** DAPI: nucleus stain. **(M,R)** Merging Nell-1 with angiogenetic markers. d, dentin; p, dental pulp; bd, blood vessel.

Cell immunofluorescence assay displayed that Nell-1 is mainly expressed in the nucleus of DPSCs and HUVECs (Figures 5A–H). HE staining and immunofluorescence were conducted to present the structure of normal pulp tissues and the distribution of Nell-1.

Double immunofluorescence of pulp tissue sections was used to observe the distribution of Nell-1, VEGF, and Flk-1 in the dental pulp (Figures 5I–R). High expression of Nell-1, VEGF, and Flk-1 was found in the odontoblasts, pulp fibroblasts, and endothelial cells of the blood vessels of the dental pulp. The merged picture showed that Nell-1 is co-expressed with VEGF and Flk-1.

### Histology and Immunohistochemistry

**FIGURE 6:**
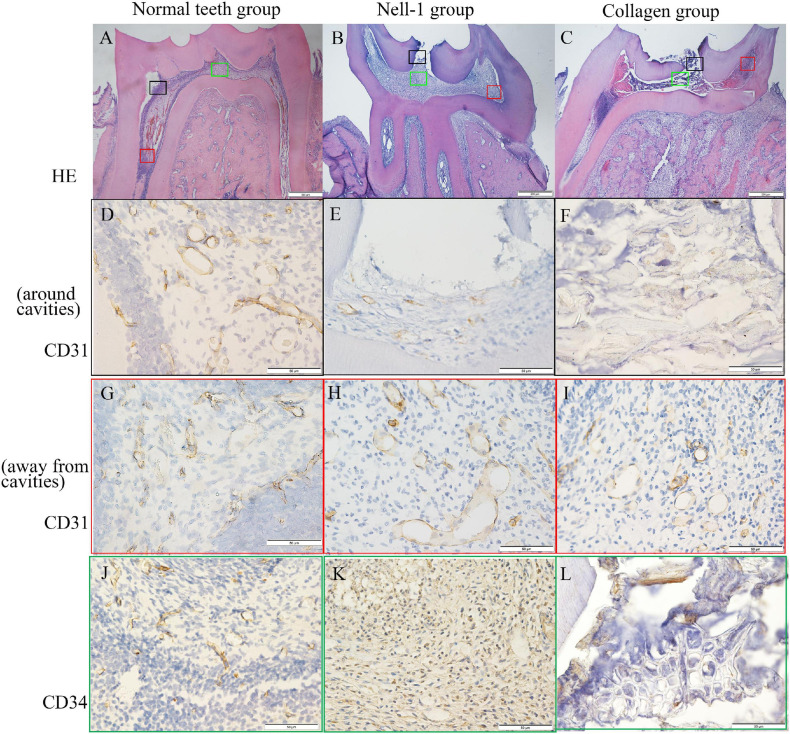
Histology and immunohistochemistry staining. **(A)** Normal teeth group. **(B)** Nell-1 group. **(C)** Collagen group. HE staining showed the cavities, inflamed tissue, and normal structure of rat pulp tissues. The staining of CD31 **(D–I)** and CD34 **(J–L)** revealed the blood vessels in pulp tissues. Nell-1 induction group had higher number of blood vessels around cavities than the collagen group, and both of them had fewer blood vessels than negative control group **(D–F,J–L)**. There was no significant difference in the area away from the cavities between three groups **(G–I)**. d, dentin; p, dental pulp; bd, blood vessel.

At week 1, inflamed tissues around cavities were discovered by HE staining (Figures 6A–C). Immunohistochemical staining of CD31 and CD34 revealed vascular lumens in pulp tissues. The collagen group had higher amount of inflammatory cell infiltration but lower numbers of blood vessels around cavities than the Nell-1 group (Figures 6E,F,K,L). Both groups had more inflammatory cell infiltration and fewer blood vessels than normal teeth group (Figures 6D–L). In addition, no significant difference in the area away from the cavities was found among the three groups (Figures 6G–I).

## Discussion

DPSCs are regarded as great stem cell sources in different generation fields because they could be easily isolated by a non-invasive method and without moral concern (Lee et al., 2019; Yamada et al., 2019). Direct co-culture of DPSCs and HUVECs was applied in this study according to the previous studies which has showed greater expression of angiogenic markers compared with DPSCs alone (Dissanayaka et al., 2012).

In this study, Nell-1 upregulated VEGF and Flk-1, which are responsible for proangiogenic properties. Flk-1 protein expression was downregulated in the co-culture group at day 3 when Nell-1 was added; however, the mechanisms are still unclear. Cell immunofluorescence staining revealed that Nell-1 is expressed in the nucleus of DPSCs and HUVECs and co-expressed with VEGF and Flk-1 in normal pulp tissues. This finding indicates that Nell-1 may have a synergistic or similar effect with VEGF and Flk-1 on promoting angiogenesis. The *in vitro* tube formation assay also confirmed Nell-1 effects on promoting angiogenesis.

The survival rate of inflamed pulp tissue is related to the surrounding angiogenesis (Saghiri et al., 2015). Immunohistochemical staining of CD31 and CD34 indicated that the number of blood vessels in the Nell-1 group is higher than that in the collagen group. This phenomenon can be explained as follows. *In vitro* experiment revealed that Nell-1 may promote angiogenesis by increasing the expression of VEGF and Flk-1 in DPSCs. Considering the co-expression of Nell-1 and angiogenetic markers, we speculate that Nell-1 may have a synergistic or similar effect to VEGF and Flk-1.

Pulp regeneration mainly includes blood vessels, nerves, and dentin. In an adult tooth, each large myelinated nerve fiber bundle surrounds a single arteriole in the root canals and pulp chambers (Steiniger et al., 2013). During early tooth formation, tooth innervation occurs through vasculogenesis (Shadad et al., 2019). In this study, we found that Nell-1 can enhance the expression of proteins and angiogenetic genes as early as day 2, which was almost the same rate as that of the odontoblastic and neural-like differentiation of DPSCs (Liu et al., 2016; Han et al., 2019). Subsequent experiments will be conducted to confirm the relevant mechanisms. Nell-1 application may be a promising strategy in dental pulp regeneration field since its contribution to dentin formation, neurogenesis and angiogenesis (Liu et al., 2016; Han et al., 2019).

Xuan et al. (2018) confirmed that human deciduous autologous tooth stem cells can regenerate the whole dental pulp and have a promising effect on pulp regeneration; however, the mechanisms are still unclear. The present study investigated the effect of Nell-1 on pulp angiogenesis and found that this molecule may promote the angiogenic differentiation of DPSCs, thus further confirming its role in pulp regeneration.

## Conclusion

Nell-1 is highly expressed in the nucleus of DPSCs and HUVECs and co-expressed with angiogenetic markers in normal pulp tissues. This molecule could enhance the angiogenic differentiation of DPSCs *in vitro* and *in vivo* and thus may be a promising drug for the regeneration of the whole pulp.

## Data Availability Statement

The original contributions presented in the study are included in the article/supplementary material, further inquiries can be directed to the corresponding author/s.

## Ethics Statement

The studies involving human participants were reviewed and approved by Institutional Review Board of School of Stomatology (No. R20180801). The patients/participants provided their written informed consent to participate in this study. The animal study was reviewed and approved by Institutional Review Board of School of Stomatology (No. D20180801).

## Author Contributions

XW and QW designed, supervised, and funded the study. ML performed the experiments and prepared the manuscript. QH, JW, and HZ performed the animal experiment. XB and YC performed the analysis. CY collected the clinical sample. All authors were actively involved with their work on this manuscript. All authors read and approved the final manuscript.

## Conflict of Interest

The authors declare that the research was conducted in the absence of any commercial or financial relationships that could be construed as a potential conflict of interest.

## Publisher’s Note

All claims expressed in this article are solely those of the authors and do not necessarily represent those of their affiliated organizations, or those of the publisher, the editors and the reviewers. Any product that may be evaluated in this article, or claim that may be made by its manufacturer, is not guaranteed or endorsed by the publisher.

## References

[B1] Aguirre A. Planell J. A. Engel E. (2010). Dynamics of bone marrow-derived endothelial progenitor cell/mesenchymal stem cell interaction in co-culture and its implications in angiogenesis. *Biochem. Biophys. Res. Commun.* 400 284–291. 10.1016/j.bbrc.2010.08.073 20732306

[B2] Allen P. Melero-Martin J. Bischoff J. (2011). Type I collagen, fibrin and PuraMatrix matrices provide permissive environments for human endothelial and mesenchymal progenitor cells to form neovascular networks. *J. Tissue Eng. Regen. Med.* 5 e74–e86. 21413157 10.1002/term.389 PMC3178449

[B3] An S. Y. Lee Y. J. Neupane S. Jun J. H. Kim J. Y. Lee Y. (2017). Effects of vascular formation during alveolar bone process morphogenesis in mice. *Histochem. Cell Biol.* 148 435–443. 10.1007/s00418-017-1584-2 28612087

[B4] Chung A. S. Ferrara N. (2011). Developmental and pathological angiogenesis. *Annu. Rev. Cell Dev. Biol.* 27 563–584. 10.1146/annurev-cellbio-092910-154002 21756109

[B5] d’Aquino R. Graziano A. Sampaolesi M. Laino G. Pirozzi G. De Rosa A. (2007). Human postnatal dental pulp cells co-differentiate into osteoblasts and endotheliocytes: a pivotal synergy leading to adult bone tissue formation. *Cell Death Differ.* 14 1162–1171. 10.1038/sj.cdd.4402121 17347663

[B6] Dissanayaka W. L. Zhan X. Zhang C. Hargreaves K. M. Jin L. Tong E. H. (2012). Coculture of dental pulp stem cells with endothelial cells enhances osteo-/odontogenic and angiogenic potential in vitro. *J. Endod.* 38 454–463. 10.1016/j.joen.2011.12.024 22414829

[B7] Fahmy-Garcia S. van Driel M. Witte-Buoma J. Walles H. van Leeuwen J. P. T. M. van Osch G. J. V. M. (2018). NELL-1, HMGB1, and CCN2 enhance migration and vasculogenesis, but not osteogenic differentiation compared to BMP2. *Tissue Eng. Part A* 24 207–218. 10.1089/ten.tea.2016.0537 28463604

[B8] Ferrara N. (2002). Role of vascular endothelial growth factor in physiologic and pathologic angiogenesis: therapeutic implications. *Semin. Oncol.* 29(6 Suppl. 16), 10–14. 10.1053/sonc.2002.37264 12516033

[B9] Ferrara N. Gerber H. P. LeCouter J. (2003). The biology of VEGF and its receptors. *Nat. Med.* 09 669–676. 10.1038/nm0603-669 12778165

[B10] Gonmanee T. Thonabulsombat C. Vongsavan K. Sritanaudomchai H. (2018). Differentiation of stem cells from human deciduous and permanent teeth into spiral ganglion neuron-like cells. *Arch. Oral. Biol.* 88 34–41. 10.1016/j.archoralbio.2018.01.011 29407749

[B11] Gronthos S. Mankani M. Brahim J. Robey P. G. Shi S. (2000). Postnatal human dental pulp stem cells in vitro and in vivo. *PNAS* 97 13625–13630. 11087820 10.1073/pnas.240309797 PMC17626

[B12] Han Q. Wang Q. Wu J. Li M. Fang Y. Zhu H. (2019). Nell-1 promotes the neural-like differentiation of dental pulp cells. *Biochem. Biophys. Res. Commun.* 513 515–521. 10.1016/j.bbrc.2019.04.028 30979495

[B13] Hilkens P. Fanton Y. Martens W. Gervois P. Struys T. Politis C. (2014). Pro-angiogenic impact of dental stem cells in vitro and in vivo. *Stem Cell Res.* 12 778–790. 10.1016/j.scr.2014.03.008 24747218

[B14] Kang Y. Kim S. Fahrenholtz M. Khademhosseini A. Yang Y. (2013). Osteogenic and angiogenic potentials of monocultured and co-cultured human-bone-marrow-derived mesenchymal stem cells and human-umbilical-vein endothelial cells on three-dimensional porous beta-tricalcium phosphate scaffold. *Acta Biomater.* 9 4906–4915. 10.1016/j.actbio.2012.08.008 22902820 PMC3508299

[B15] Karbanova J. Soukup T. Suchanek J. Pytlík R. Corbeil D. Mokrý J. (2011). Characterization of dental pulp stem cells from impacted third molars cultured in low serum-containing medium. *Cells Tissues Organs* 193 344–365. 10.1159/000321160 21071916

[B16] Lee Y. C. Chan Y. H. Hsieh S. C. Lew W. Z. Feng S. W. (2019). Comparing the osteogenic potentials and bone regeneration capacities of bone marrow and dental pulp mesenchymal stem cells in a rabbit calvarial bone defect model. *Int. J. Mol. Sci.* 20:5015. 10.3390/ijms20205015 31658685 PMC6834129

[B17] Leopold B. Strutz J. Weiß E. Gindlhuber J. Birner-Gruenberger R. Hackl H. (2019). Outgrowth, proliferation, viability, angiogenesis and phenotype of primary human endothelial cells in different purchasable endothelial culture media: feed wisely. *Histochem. Cell Biol.* 152 377–390. 10.1007/s00418-019-01815-2 31541300 PMC6842357

[B18] Li C. Zhang X. Zheng Z. Nguyen A. Ting K. Soo C. (2019). Nell-1 is a key functional modulator in osteochondrogenesis and beyond. *J. Dent. Res.* 98 1458–1468. 10.1177/0022034519882000 31610747 PMC6873286

[B19] Li D. Zou X. Y. El-Ayachi I. Romero L. O. Yu Z. Iglesias-Linares A. (2019). Human dental pulp stem cells and gingival mesenchymal stem cells display action potential capacity in vitro after neuronogenic differentiation. *Stem Cell Rev. Rep.* 15 67–81. 10.1007/s12015-018-9854-5 30324358 PMC6358481

[B20] Liu M. Wang Q. Tang R. Cao R. Wang X. (2016). Nel-like molecule 1 contributes to the odontoblastic differentiation of human dental pulp cells. *J. Endod.* 42 95–100. 10.1016/j.joen.2015.08.024 26456257

[B21] Liu M. Zhao L. Hu J. Wang L. Li N. Wu D. (2018). Endothelial cells and endothelin1 promote the odontogenic differentiation of dental pulp stem cells. *Mol. Med. Rep.* 18 893–901. 29845193 10.3892/mmr.2018.9033 PMC6059721

[B22] Moioli E. K. Clark P. A. Chen M. Dennis J. E. Erickson H. P. Gerson S. L. (2008). Synergistic actions of hematopoietic and mesenchymal stem/progenitor cells in vascularizing bioengineered tissues. *PLoS One* 3:e3922. 10.1371/journal.pone.0003922 19081793 PMC2597748

[B23] Rao R. R. Peterson A. W. Ceccarelli J. Putnam A. J. Stegemann J. P. (2012). Matrix composition regulates three-dimensional network formation by endothelial cells and mesenchymal stem cells in collagen/fibrin materials. *Angiogenesis* 15 253–264. 10.1007/s10456-012-9257-1 22382584 PMC3756314

[B24] Rombouts C. Giraud T. Jeanneau C. About I. (2017). Pulp vascularization during tooth development, regeneration, and therapy. *J. Dent. Res.* 96 137–144. 10.1177/0022034516671688 28106505

[B25] Saghiri M. A. Asatourian A. Sorenson C. M. Sheibani N. (2015). Role of angiogenesis in endodontics: contributions of stem cells and proangiogenic and antiangiogenic factors to dental pulp regeneration. *J. Endod.* 41 797–803. 10.1016/j.joen.2014.12.019 25649306 PMC5223201

[B26] Shadad O. Chaulagain R. Luukko K. Kettunen P. (2019). Establishment of tooth blood supply and innervation is developmentally regulated and takes place through differential patterning processes. *J. Anat.* 234 465–479. 10.1111/joa.12950 30793310 PMC6422796

[B27] Steiniger B. S. Bubel S. Bockler W. Lampp K. Seiler A. Jablonski B. (2013). Immunostaining of pulpal nerve fibre bundle/arteriole associations in ground serial sections of whole human teeth embedded in technovit(R) 9100. *Cells Tissues Organs* 198 57–65. 10.1159/000351608 23797205

[B28] Stevens A. Zuliani T. Olejnik C. LeRoy H. Obriot H. Kerr-Conte J. (2008). Human dental pulp stem cells differentiate into neural crest-derived melanocytes and have label-retaining and sphere-forming abilities. *Stem Cells Dev.* 17 1175–1184. 10.1089/scd.2008.0012 18393638

[B29] Tang R. Wang Q. Du J. Yang P. Wang X. (2013). Expression and localization of Nell-1 during murine molar development. *J. Mol. Histol.* 44 175–181. 10.1007/s10735-012-9472-5 23264108

[B30] Ting K. Vastardis H. Mulliken J. B. Soo C. Tieu A. Do H. (1999). Human Nell-1 expressed in unilateral coronal synostosis. *J. Bone Miner. Res.* 14 80–89. 10.1359/jbmr.1999.14.1.80 9893069

[B31] Xuan K. Li B. Guo H. Sun W. Kou X. He X. (2018). Deciduous autologous tooth stem cells regenerate dental pulp after implantation into injured teeth. *Sci. Transl. Med.* 10:eaaf3227. 10.1126/scitranslmed.aaf3227 30135248

[B32] Yamada Y. Nakamura-Yamada S. Kusano K. Baba S. (2019). Clinical potential and current progress of dental pulp stem cells for various systemic diseases in regenerative medicine: a concise review. *Int. J. Mol. Sci.* 20:1132. 10.3390/ijms20051132 30845639 PMC6429131

[B33] Zhang X. Zara J. Siu R. K. Ting K. Soo C. (2010). The role of Nell-1, a growth factor associated with craniosynostosis, in promoting bone regeneration. *J. Dent. Res.* 89 865–878. 10.1177/0022034510376401 20647499 PMC2959101

